# Sex differences in the impact of sedentary time on early-onset metabolic multimorbidity: evidence from a study of over 20,000 young adults

**DOI:** 10.1186/s13293-026-00831-x

**Published:** 2026-01-28

**Authors:** Chunjun Li, Mianzhi Zhang, Li Zhang, Fenghua Guo, Binbin Zhang, Shuo Chen, Yujie Niu, Feng Liu, Minying Zhang

**Affiliations:** 1 School of Medicine, Nankai University, 94, Weijin Road, Tianjin, 300071 China; 2 The First Affiliated Hospital, Nankai University, Tianjin, 300121 China; 3 Tianjin Hospital of Integrated Traditional Chinese and Western Medicine, Tianjin, 300100 China; 4 Dongfang Hospital, Beijing University of Chinese Medicine, Beijing, 100078 China; 5 Tianjin Institute of Nephropathy of Traditional Chinese Medicine, Tianjin, 300120 China; 6 Tianjin First Central Hospital, Tianjin, 300192 China; 7 Beijing Physical Examination Center, Beijing, 100029 China; 8 Department of Occupational Health and Environmental Health, Hebei Medical University, Shijiazhuang, 050017 China

**Keywords:** Sedentary time, Metabolic multimorbidity, Young adults, Sex-specific association, Interaction

## Abstract

**Background:**

Both sedentary lifestyles and metabolic multimorbidity are experiencing a rising global prevalence, representing growing public health concerns. Although the detrimental effects of sedentary behavior on metabolic diseases are well-documented, the association between sedentary time and early-onset metabolic multimorbidity, especially potential sex-specific differences in this association, remains unexplored. This study aimed to investigate the sex-specific effects of sedentary time on the prevalence of early-onset metabolic multimorbidity.

**Methods:**

We recruited 21,444 young adults (18–45 years; 50.6% women) and assessed their sedentary time using the International Physical Activity Questionnaire-Short Form (IPAQ-SF). Metabolic multimorbidity was defined as the coexistence of at least two of the following conditions: obesity, hypertension, dyslipidemia, diabetes, and non-alcoholic fatty liver disease. Logistic regression models were used to evaluate the sex-specific association between sedentary time and metabolic multimorbidity. Subgroup analyses by sociodemographic characteristics and lifestyle factors were performed to identify potential interactions and susceptible subpopulations. Sensitivity analysis was conducted to assess the robustness of the results.

**Results:**

The overall prevalence of metabolic multimorbidity was 25.35%, with a significantly higher prevalence in males than in females (40.72% vs. 10.35%), despite a higher proportion of females reporting longer sedentary time. In males, sedentary time exhibited a linear positive association with the increased odds of metabolic multimorbidity, whereas no such association was observed in females. Males reporting 6-<8 and ≥ 8 h/day sedentary time had 22% (95% CI 1.01–1.47) and 26% (95% CI 1.05–1.50) higher odds of metabolic multimorbidity, respectively, compared to those reporting < 4 h/day sedentary time (*P*_for trend_ = 0.019). Education level, smoking status and physical activity level were found to modify the association between sedentary time and metabolic multimorbidity.

**Conclusion:**

Longer sedentary time is associated with increased odds of early-onset metabolic multimorbidity in men, but this association is not observed in women. These findings suggest that targeted, sex-specific interventions may reduce metabolic risk and improve long-term outcomes, highlighting the need to consider sex differences in both prevention strategies and expected health benefits.

**Supplementary Information:**

The online version contains supplementary material available at 10.1186/s13293-026-00831-x.

## Abstract (plain language summary)

This study examines how sitting for a long time affects the prevalence of multiple metabolic diseases early in life, focusing on differences between men and women. Researchers analyzed data from over 21,000 young adults aged 18 to 45, finding that men who sat for more than 6 h a day showed a higher prevalence of metabolic diseases like obesity, high blood pressure, and diabetes. Specifically, sitting 6–8 h daily was associated with a 22% higher prevalence of these conditions, and those sitting over 8 h showed a 26% higher prevalence. However, no such link was found in women. The study highlights that while women reported more sedentary time, men exhibited a stronger association between sitting duration and metabolic disease prevalence. Factors like education level, smoking, and physical activity influenced these associations differently for men and women. The findings suggest that reducing sedentary behavior could help lower the prevalence of metabolic diseases, especially in men. This research underscores the importance of considering gender differences in health interventions. It also points out the need for more studies to understand why men and women respond differently to sedentary lifestyles. Overall, the study emphasizes the need for targeted strategies to combat the health risks associated with longer sitting time, particularly among young men.

**Supplementary Information:**

The online version contains supplementary material available at 10.1186/s13293-026-00831-x.

## Highlights

- Men, but not women, exhibit a significant association between longer sedentary time and an elevated prevalence of metabolic multimorbidity.
- Men who are sedentary for ≥ 6 h/day experience a 22–26% higher odds of metabolic multimorbidity compared to those sedentary for < 4 h/day.
- No significant link was found between sedentary time and metabolic diseases in women.
- Education level, smoking status, and physical activity levels modify the association between sedentary time and metabolic multimorbidity in men, indicating gender-specific influences.
- The study underscores the necessity for tailored interventions to mitigate sedentary behavior and enhance metabolic health, particularly among men.

**Supplementary Information:**

The online version contains supplementary material available at 10.1186/s13293-026-00831-x.

## Introduction

The elevated prevalence of metabolic diseases has emerged as a critical public health challenge [1], contributing to a substantial clinical and economic burden worldwide [2]. Metabolic multimorbidity, as one of the most prevalent patterns of multiple chronic conditions [3], is associated with poor long-term prognoses, including disability, functional decline, and increased mortality [4, 5]. Although metabolic diseases have conventionally been considered prevalent among middle-aged and older populations, recent reports have highlighted a rising incidence of metabolic diseases in younger adults [6–10]. Growing evidence indicates that early-onset metabolic diseases are closely linked to vascular disease [11], cancer [12], and premature mortality [13], with an even stronger association when multiple metabolic conditions coexist [14]. However, there is a notable paucity of research on early-onset metabolic multimorbidity in young adults.

The advent of the information age has paradoxically fostered the proliferation of sedentary lifestyles, which are characterized by high levels of screen time and prolonged sitting, particularly among young populations [15]. In recent years, the global prevalence of sedentary behavior has surged, largely due to computer-based occupations [15], sedentary leisure activities (e.g., television watching, video gaming, phone calls, or texting) [16] and motorized transportation [17]. Moreover, accumulating evidence indicates that excessive sedentary time is associated with adverse health outcomes [18]. A dose-response meta-analysis of 58 studies demonstrated that each additional hour of sedentary behavior per day was linked to a 5% increased risk of developing type 2 diabetes (T2D) [18–20]. A cross-sectional study from Finland reported the total sedentary time contributed to an elevated risk of cardiovascular disease [19], while a prospective cohort study in the United States highlighted a strong correlation between prolonged sedentary time and increased cancer mortality [20]. Nevertheless, the association of sedentary time and early-onset metabolic multimorbidity in the young adults remains unexplored.

Substantial evidence indicates that biological sex exerts profound and multifaceted influences on the pathophysiological mechanisms, clinical phenotypes, disease trajectories, and therapeutic responses across a broad spectrum of medical conditions. Research has elucidated sexually dimorphic metabolic profiles, wherein women consistently manifest higher degrees of insulin resistance, a physiological state that is partially attributable to their comparatively lower skeletal muscle mass and higher adipose tissue deposition relative to age-matched males [21, 22]. The fundamental relationship between adipose tissue distribution patterns and insulin sensitivity has been firmly established in metabolic physiology [23]. Emerging evidence indicates that adipose tissue distribution pattern exerts a disproportionately greater disruptive effect on glucose homeostasis in the female sex, suggesting underlying sex-specific metabolic regulatory mechanisms [22]. Sex differences in metabolic diseases are well-documented. For instance, prevalence rates of metabolic disorders are higher in adolescent girls, shift in favor of males during middle age, and become comparable in older populations [24–26]. Women with T2D typically demonstrate poorer cardiopulmonary fitness than their male counterparts and contribute disproportionately more to the overall cardiovascular disease burden within the diabetic population [27].

Notable sex differences also exist in physical activity (PA) patterns. Large-scale population-based studies indicate that adult women engage in markedly lower volumes of total PA, with particular deficits in moderate-to-vigorous intensity exercise participation [28]. Experimental research has shown that interrupting prolonged sitting with intermittent walking confers beneficial effects on glucose metabolism. Notably, these beneficial metabolic responses demonstrate significant sexual dimorphism, with mixed-gender samples demonstrating greater postprandial glucose improvements than female-exclusive groups [29]. These findings provide compelling evidence that males may exhibit heightened susceptibility to the deleterious metabolic consequences of chronic sedentary behavior.

The contribution of sedentary behavior to the global burden of metabolic diseases and the critical moderating role of biological sex in this relationship are well-established. However, critical evidence gaps remain. First, despite the established link between sedentary behavior and individual metabolic diseases, evidence regarding its association with early-onset metabolic multimorbidity, a cluster of conditions with profound long-term health implications, is notably scarce, especially in young adults. Second, whether these associations differ significantly by sex​ is largely unexplored, despite compelling biological and behavioral rationales for such differences.

To address these gaps, we utilized data from the Beijing-Tianjin-Hebei Medical Examination Cohort (BTH-MEC) to conduct, to our knowledge, the first large-scale study​ specifically designed to quantify the sex-specific association between sedentary time and early-onset metabolic multimorbidity among adults under 45 years of age. Furthermore, we sought to examine whether key sociodemographic and lifestyle factors (e.g., education, physical activity) modify this association.

## Methods

### Study design and participants

**Fig. 1:**
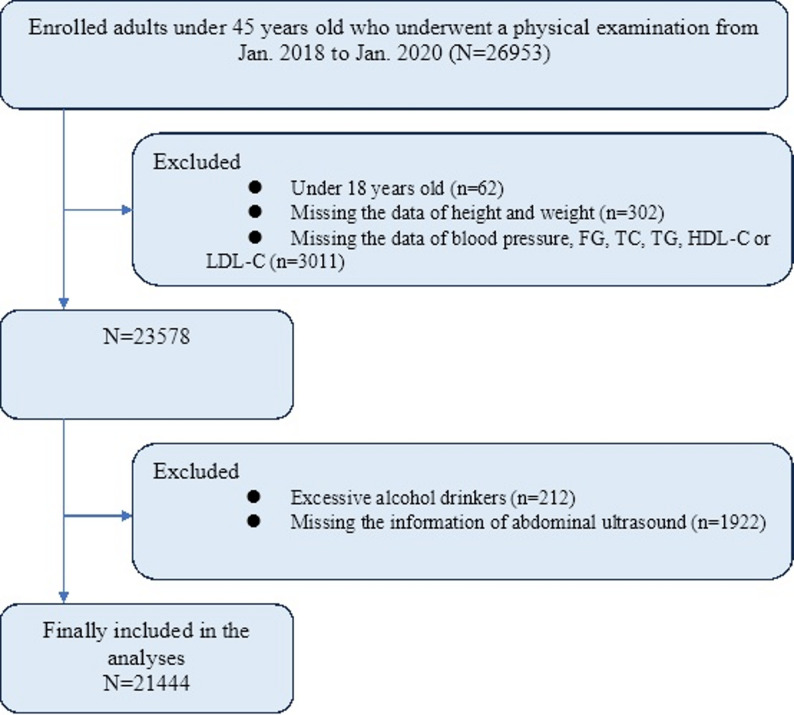
The recruitment of the study population

The data used in this analysis were collected as part of the baseline data from the BTH-MEC, a National Key Research & Development Program of China. Participants were recruited through multi-stage stratified cluster sampling at eight selected medical examination centers in the Beijing-Tianjin-Hebei region from January 2018 to January 2020. A total of 26,953 participants under 45 years of age completed a standard questionnaire, anthropometric measurements, and biochemical tests. After excluding participants under 18 years of age (*n* = 62), those missing data on height, weight, blood pressure (BP), fasting glucose (FG), total cholesterol (TC), triglycerides (TG), high-density lipoprotein cholesterol (HDL-C), or low-density lipoprotein cholesterol (LDL-C) (*n* = 3313), those with excessive alcohol consumption (*n* = 212), and those missing abdominal ultrasound information (*n* = 1922), a total of 21,444 participants were included in the current study (Fig. 1). The study protocol was approved by the Medical Ethics Committee of Nankai University (NKUIRB2016063). All participants provided written informed consent.

## Data collection

### Assessment of sedentary time

Sedentary time was assessed using the International Physical Activity Questionnaire-Short Form (IPAQ-SF). Participants were asked to self-report the following question: During the last 7 days, how much time did you spend sitting, reclining, or lying down each weekday? The Sedentary time includes the time spent at work, at home while doing coursework, and during leisure activities such as working at a desk or computer, visiting friends, driving, reading, writing, watching television, playing games, commuting, using the Internet, or listening to music during waking hours. For analysis, sedentary time was categorized into four groups: <4, 4–<6, 6–<8, and ≥ 8 h/day.

### Assessment of metabolic multimorbidity

In this study, metabolic multimorbidity was defined as the coexistence of at least two of the following metabolic conditions: hypertension, dyslipidemia, T2D, obesity, or non-alcoholic fatty liver disease (NAFLD), based on clinical diagnosis or ongoing pharmacological treatment at baseline, according to Chinese Guidelines. Diagnostic criteria for metabolic disease differ between Europe/America and China. This divergence is fundamentally attributed to the expert consensus on ethnic disparities, whereby Asians are more susceptible to visceral adiposity and metabolic complications at lower anthropometric values. However, employing China-specific criteria is more appropriate for our target population and carries more substantial public health implications.

Hypertension was diagnosed according to the Chinese clinical practice guideline for the management of hypertension (2024 edition), which defined hypertension as SBP ≥ 140 mmHg and/or DBP ≥ 90 mmHg, or self-reported history of diagnosed hypertension [30]. Dyslipidemia was diagnosed according to the 2023 edition of the Chinese guideline for the management of lipid in adults, which defined dyslipidemia as TG ≥ 2.26 mmol/L and/or TC ≥ 6.22 mmol/L and/or LDL-C ≥ 4.14 mmol/L, HDL-C ≤ 1.04 mmol/L, or self-reported history of diagnosed dyslipidemia [31]. T2D was diagnosed according to the 2024 edition of the guidelines for the prevention and control of T2D in China, which defined T2D as FG ≥ 7.0 mmol/L and/or 2-h postprandial blood glucose in oral glucose tolerance test (OGTT) ≥ 11.1 mmol/L, or self-reported previous diagnosis of T2D [32]. Asian-specific cut-points were used to define obesity as BMI ≥ 28.0 kg/m^2^ [33]. NAFLD was diagnosed according to the ultrasonic criteria suggested by the Chinese Medical Association [34], which define NAFLD based on the following items: (1) diffuse enhancement of near-field echo in the hepatic region (stronger than in the kidney and spleen regions) and gradual attenuation of far-field echo; (2) unclear display of intra-hepatic lacuna structure; (3) mild to moderate hepatomegaly with a round and blunt border; (4) color Doppler ultrasonography shows a reduction in the blood flow signal in the liver, or it is even difficult to display, though the distribution of blood flow is normal. NAFLD was diagnosed if item 1 and at least one of items 2–4 were observed.

### Assessment of covariates

Information on demographics, medical history, and lifestyle was collected using a standardized questionnaire administered by trained staff, including sex, age, occupation (professional technologists, civil servants, or others), marital status (married, single, or divorced/widowed), education level (senior high school or below, college education, postgraduate or above), family history of metabolic diseases, smoking status (never smoked, former smoker, or current smoker), alcohol consumption (non-drinker, former alcohol drinker, or current drinker), and sleep duration. PA-related information was collected using the IPAQ-SF. The metabolic equivalent of task (MET) values for vigorous, moderate, and low-intensity activities was defined as ​8.0, 4.0, and 3.3 METs, respectively (where ​1 MET = 1 kcal·kg⁻¹·h⁻¹). Each type of PA was assigned its corresponding MET value. Further, the PA level was calculated by multiplying the METs, duration (hours), and frequency. For individuals engaging in multiple types of PA, weighted MET values were computed based on the ​total weekly time spent on each activity. Subsequently, participants were categorized into ​three PA intensity groups: low (1–600 METs), moderate (600–3000 METs), and high (> 3000 METs).

Prior to the face-to-face interview, venous blood samples were collected after 8–12 h of fasting. FG, TC, TG, HDL-C, and LDL-C were measured using the Hitachi 7600 automated analyzer (Hitachi, Inc., Tokyo, Japan). Weight and height were measured without shoes using a calibrated stadiometer (GL-310, Seoul, Korea). Body mass index (BMI) was calculated as weight in kilograms divided by height in meters squared (kg/m²). Systolic blood pressure (SBP) and diastolic blood pressure (DBP) were measured using a sphygmomanometer (Kenz-AC OSC, Japan) while the participant was seated. All measurements were performed by qualified medical professionals.

### Statistical analysis

All variables included in the analysis were categorical, with those originally non-categorical having been converted into categorical form. The baseline characteristics of participants were calculated and compared across sedentary time categories. We used the Cochran-Armitage test to assess overall trend across ordered sedentary categories. The Chi-square test was used to compare the prevalence of metabolic multimorbidity between males and females. Multivariate logistic regression analyses were performed for the total population, as well as separately for males and females to calculate the odds ratios (OR) with 95% confidence intervals (95% CI) for metabolic multimorbidity according to sedentary time category. Four models were applied: Model 1 was unadjusted; Model 2 was adjusted for age, occupation, marital status, and education level; Model 3 was further adjusted for family history of metabolic diseases; and Model 4 was further adjusted for smoking status, alcohol consumption, PA level, and sleep duration. In addition, the interaction term for sex and sedentary time was included in Model 4 for the total population.

In addition, to examine whether potential effect modifiers related to metabolic multimorbidity, such as age (< 35 years and ≥ 35 years), education level (high school or below, college and postgraduate or above), smoking status (nonsmoker, current smoker and former smoker), alcohol consumption (non-drinker, former drinker, and current drinker), or PA level (low, moderate, and high), interact with sedentary time in the association between sedentary time and metabolic multimorbidity, we conducted subgroup analyses stratified by these variables. We also included interaction terms between sedentary time and each of these variables in the fully adjusted regression models.

We also conducted sensitivity analyses to further assess the robustness and internal validity of our findings. To minimize potential confounding bias resulting from lifestyle modifications (such as reduced sedentary time or increased physical activity) that may occur after the diagnosis of chronic diseases, we excluded participants who had already been clinically diagnosed with hypertension, diabetes, hyperlipidemia, or NAFLD, or who were receiving pharmacological treatment for these chronic conditions at baseline. We then performed a fully adjusted logistic regression analysis to reassess the impact of sedentary time on the odds of metabolic multimorbidity.

All analyses were conducted using R version 4.0.5 software (R Foundation for Statistical Computing, Vienna, Austria) and ECharts (version: 5.1.2, https://echarts.apache.org/en/index.html) were used for data visualization. A two-sided P-value < 0.05 was considered statistically significant.

## Results

### Baseline characteristics of the participants

**Fig. 2:**
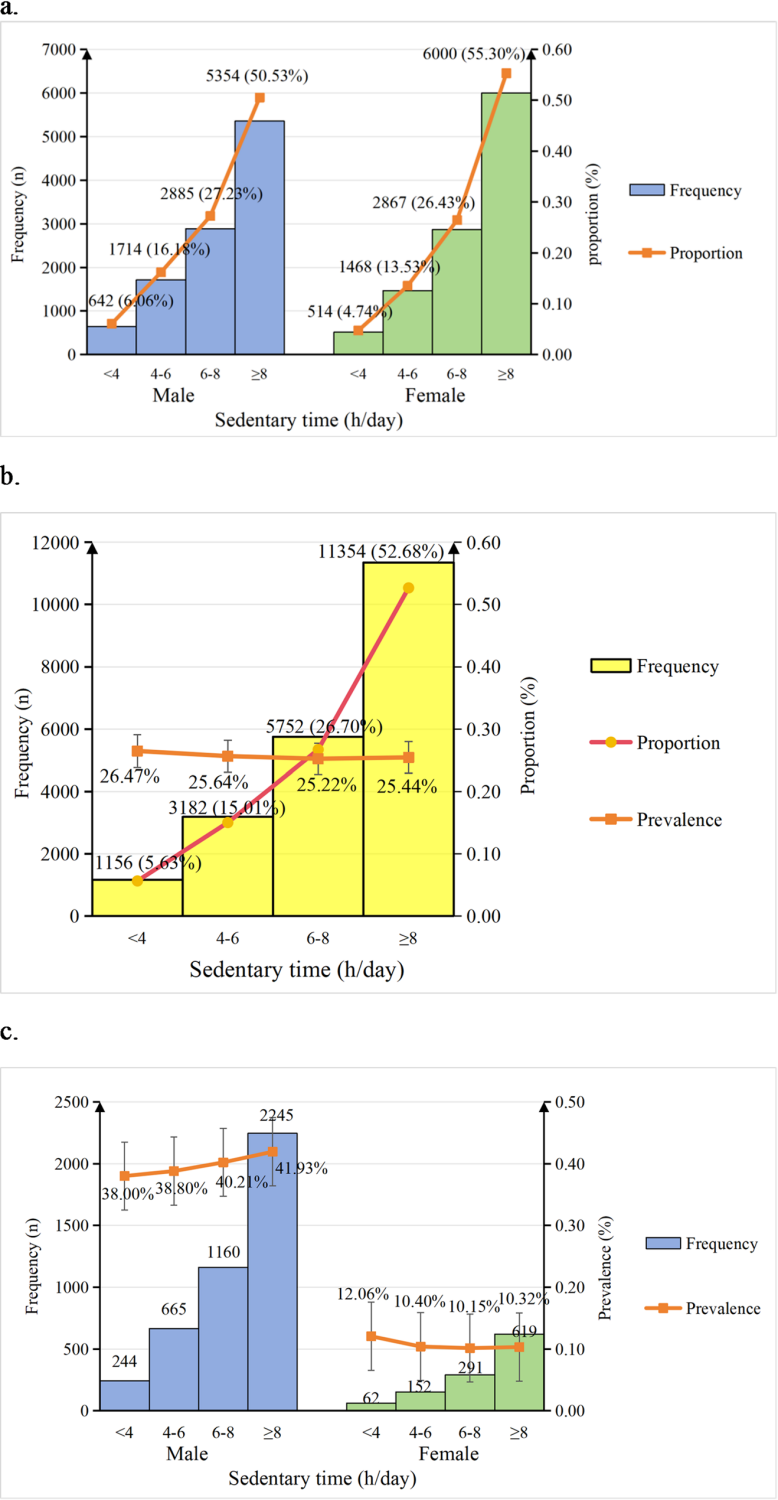
Distribution of sedentary time categories and the associated prevalence of metabolic multimorbidity in the total population and stratified by sex. (**a**) Distribution of sedentary time categories by sex. (**b**) Distribution of sedentary time categories and the associated prevalence of metabolic multimorbidity in the total population. (**c**) Sex-specific prevalence of metabolic multimorbidity across sedentary time categories. h/day, hours per day

This analysis included 21,444 adults (50.6% female) aged 18 to 45 years. Detailed characteristics stratified by sedentary time categories are presented in Table 1. A higher proportion of women reported longer sedentary time (≥ 4 h/day) compared to men (*P* < 0.001). Among men, the proportions of participants reporting < 4, 4-<6, 6-<8, and ≥ 8 h/day of sedentary time were 6.06%, 16.18%, 27.23%, and 50.53%, respectively. The corresponding proportions among women were 4.74%, 13.53%, 26.43%, and 55.30% (Fig. 2a).

**Table 1:** Baseline characteristics of study participants

Characteristics	Sedentary behavior time (males)				*P*_for trend_	Sedentary behavior time (females)				*P*_for trend_
	< 4 h/day	4-<6 h/day	6-<8 h/day	≥ 8 h/day		< 4 h/day	4-<6 h/day	6-<8 h/day	≥ 8 h/day	
Number	642	1714	2885	5354		514	1468	2867	6000	
**Age (years)**										
18–25	71 (10.69)	147 (22.14)	162 (24.4)	284 (42.77)	0.375	48 (5.07)	190 (20.08)	252 (26.64)	456 (48.21)	0.012
26–30	131 (5.67)	377 (16.33)	604 (26.16)	1197 (51.84)		89 (3.73)	330 (13.82)	594 (24.87)	1375 (57.58)	
31–35	134 (4.80)	419 (15)	778 (27.86)	1462 (52.35)		103 (3.57)	325 (11.26)	714 (24.74)	1744 (60.43)	
36–40	148 (5.26)	440 (15.65)	794 (28.24)	1430 (50.85)		146 (5.31)	347 (12.61)	760 (27.62)	1499 (54.47)	
41–45	158 (7.83)	331 (16.41)	547 (27.12)	981 (48.64)		128 (6.82)	276 (14.71)	547 (29.14)	926 (49.33)	
**Occupation**										
Professional	149 (3.74)	541 (13.58)	1179 (29.6)	2114 (53.08)	< 0.001	167 (3.34)	736 (14.71)	1385 (27.68)	2716 (54.28)	< 0.001
Civil servants	96 (3.34)	352 (12.25)	890 (30.97)	1536 (53.44)		61 (2.23)	198 (7.25)	707 (25.90)	1764 (64.62)	
Other	397 (10.62)	821 (21.96)	816 (21.83)	1704 (45.59)		286 (9.18)	534 (17.14)	775 (24.88)	1520 (48.80)	
**Marital status**										
Married	470 (5.65)	1335 (16.06)	2322 (27.93)	4186 (50.35)	0.111	417 (5.11)	1049 (12.86)	2172 (26.62)	4522 (55.42)	0.776
Single	159 (7.40)	363 (16.90)	524 (24.39)	1102 (51.31)		91 (3.56)	393 (15.37)	667 (26.09)	1406 (54.99)	
Divorced/widowed	13 (9.70)	16 (11.94)	39 (29.11)	66 (49.25)		6 (4.55)	26 (19.769)	28 (21.21)	72 (54.55)	
**Education level**										
High school or below	267 (14.54)	419 (22.82)	336 (18.30)	814 (44.34)	< 0.001	199 (24.94)	210 (26.32)	156 (19.55)	233 (29.19)	< 0.001
College education	339 (5.28)	1121 (17.47)	1864 (29.06)	3091 (48.18)		258 (3.78)	967 (14.15)	1862 (27.25)	3747 (54.83)	
Postgraduate or above	36 (1.54)	174 (7.42)	685 (29.22)	1449 (61.82)		57 (1.77)	291 (9.05)	849 (26.39)	2020 (62.79)	
**Family history of metabolic diseases**										
No	353 (6.34)	971 (17.43)	1575 (28.27)	2672 (47.96)	< 0.001	317 (5.98)	793 (14.96)	1385 (26.13)	2806 (52.93)	< 0.001
Unknown	99 (16.26)	140 (22.99)	157 (25.78)	213 (34.98)		34 (6.12)	105 (18.88)	152 (27.34)	265 (47.66)	
Yes	190 (4.30)	603 (13.66)	1153 (26.12)	2469 (55.92)		163 (3.27)	570 (11.42)	1330 (26.64)	2929 (58.67)	
**Smoking status**										
Never smoked	396 (5.98)	991 (14.96)	1853 (27.97)	3386 (51.09)	0.001	504 (4.74)	1432 (13.46)	2812 (26.43)	5891 (55.37)	0.287
Former smoker	17 (4.24)	61 (15.21)	100 (24.94)	223 (55.61)		2 (6.25)	5 (15.63)	10 (31.25)	15 (46.88)	
Current smoker	229 (6.42)	662 (18.55)	932 (26.12)	1745 (48.91)		8 (4.49)	31 (17.42)	45 (25.28)	94 (52.81)	
**Alcohol intake**										
Never	397 (6.24)	918 (14.43)	1661 (26.12)	3384 (53.21)	< 0.001	485 (4.71)	1387 (13.47)	2705 (26.27)	5720 (55.55)	0.056
Former alcohol user	12 (8.82)	24 (17.65)	37 (27.21)	63 (46.32)		2 (8.33)	1 (4.17)	7 (29.17)	14 (58.33)	
Current alcohol user	233 (5.68)	772 (18.83)	1187 (28.96)	1907 (46.52)		27 (5.11)	80 (15.15)	155 (29.36)	266 (50.38)	
**PA level**										
Low	241 (7.40)	446 (13.70)	757 (23.25)	1812 (55.65)	< 0.001	232 (5.85)	486 (12.26)	898 (22.66)	2347 (59.22)	< 0.001
Moderate	234 (5.20)	668 (14.83)	1274 (28.29)	2327 (51.68)		164 (3.38)	650 (13.40)	1334 (27.49)	2704 (55.73)	
High	167 (5.89)	600 (21.16)	854 (30.11)	1215 (42.84)		118 (5.80)	332 (16.32)	635 (31.22)	949 (46.66)	
**Sleep time**										
4–6 h/day	54 (4.21)	186 (14.51)	311 (24.26)	731 (57.02)	< 0.001	52 (5.88)	129 (14.59)	183 (20.71)	520 (58.82)	0.022
7–8 h/day	495 (6.07)	1307 (16.04)	2238 (27.46)	4110 (50.43)		369 (4.51)	1079 (13.18)	2178 (26.6)	4561 (55.71)	
9–11 h/day	93 (8.00)	221 (19.00)	336 (28.89)	513 (44.11)		93 (5.23)	260 (14.62)	506 (28.46)	919 (51.69)	
**T2D**										
No	621 (6.07)	1658 (16.22)	2789 (27.28)	5156 (50.43)	0.319	506 (4.73)	1444 (13.49)	2828 (26.42)	5927 (55.37)	0.198
Yes	21 (5.66)	56 (15.09)	96 (25.88)	198 (53.37)		8 (5.56)	24 (16.67)	39 (27.08)	73 (50.69)	
**Hypertension**										
No	460 (5.82)	1260 (15.94)	2186 (27.65)	3999 (50.59)	0.141	457 (4.54)	1356 (13.46)	2647 (26.27)	5616 (55.74)	< 0.001
Yes	182 (6.77)	454 (16.88)	699 (25.99)	1355 (50.37)		57 (7.37)	112 (14.49)	220 (28.46)	384 (49.68)	
**Obesity**										
No	491 (6.18)	1285 (16.19)	2185 (27.52)	3978 (50.11)	0.189	444 (4.40)	1351 (13.38)	2687 (26.62)	5613 (55.60)	< 0.001
Yes	151 (5.69)	429 (16.15)	700 (26.36)	1376 (51.81)		70 (9.28)	117 (15.52)	180 (23.87)	387 (51.33)	
**Dyslipidemia**										
No	470 (6.79)	1149 (16.59)	1895 (27.36)	3412 (49.26)	< 0.001	471 (4.87)	1313 (13.58)	2558 (26.45)	5328 (55.1)	0.078
Yes	172 (4.69)	565 (15.40)	990 (26.98)	1942 (52.93)		43 (3.65)	155 (13.15)	309 (26.20)	672 (57.00)	
**NAFLD**										
No	399 (6.90)	994 (17.19)	1556 (26.90)	2835 (49.01)	< 0.001	433 (4.74)	1263 (13.82)	2430 (26.58)	5016 (54.87)	0.049
Yes	243 (5.05)	720 (14.97)	1329 (27.62)	2519 (52.36)		81 (4.75)	205 (12.01)	437 (25.60)	984 (57.64)	
**Metabolic Multimorbidity**										
No	398 (6.34)	1049 (16.7)	1725 (27.46)	3109 (49.5)	0.005	452 (4.65)	1317 (13.54)	2576 (26.49)	5381 (55.33)	0.503
Yes	244 (5.66)	665 (15.41)	1160 (26.89)	2245 (52.04)		62 (5.52)	151 (13.45)	291 (25.91)	619 (55.12)	
**BMI (kg/m** ^**2**^ **)**	25.52 ± 4.00	25.77 ± 4.03	25.72 ± 3.81	25.86 ± 3.98	0.059	23.67 ± 3.67	22.71 ± 3.39	22.56 ± 3.40	22.40 ± 3.42	< 0.001
**FG (mmol/L)**	5.25 ± 1.02	5.26 ± 1.20	5.21 ± 1.19	5.22 ± 1.10	0.351	4.86 ± 0.97	4.94 ± 0.70	4.95 ± 0.74	4.94 ± 0.80	0.019
**SBP (mmHg)**	126.79 ± 15.47	125.70 ± 15.29	124.85 ± 14.68	124.27 ± 14.80	< 0.001	115.99 ± 15.35	113.47 ± 13.89	113.45 ± 14.01	112.35 ± 13.28	< 0.001
**DBP (mmHg)**	79.96 ± 12.32	79.14 ± 12.01	79.08 ± 11.24	79.57 ± 11.28	0.401	72.85 ± 11.58	71.60 ± 9.85	71.88 ± 10.24	71.43 ± 9.90	0.005
**TC (mmol/L)**	4.67 ± 0.91	4.73 ± 0.92	4.75 ± 0.89	4.79 ± 0.91	0.002	4.50 ± 0.86	4.50 ± 0.85	4.59 ± 0.84	4.58 ± 0.84	0.008
**TG (mmol/L)**	1.30 (0.86,1.97)	1.39 (0.93,2.09)	1.40 (0.95,2.14)	1.43 (0.96,2.12)	0.004	0.88 (0.61,1.18)	0.86 (0.62,1.24)	0.87 (0.66,1.21)	0.88 (0.66,1.26)	0.008
**HDL-C (mmol/L)**	1.20 ± 0.27	1.21 ± 0.28	1.22 ± 0.27	1.24 ± 0.28	< 0.001	1.40 ± 0.30	1.49 ± 0.36	1.50 ± 0.35	1.51 ± 0.36	< 0.001
**LDL-C (mmol/L)**	2.91 ± 0.70	2.94 ± 0.69	2.91 ± 0.70	2.96 ± 0.74	0.215	2.73 ± 0.62	2.68 ± 0.68	2.73 ± 0.68	2.72 ± 0.68	0.729

Categorical data are presented as n (%). Continuous variables are presented as medians with interquartile ranges (IQR) for skewed data, and as means with standard deviations (SD) for normally distributed data

PA, physical activity level; T2D, type 2 diabetes; NAFLD, nonalcoholic fatty liver disease; BMI, body mass index; FG, fasting glucose; SBP, systolic blood pressure; DBP, diastolic blood pressure; TC, total cholesterol; TG, triglyceride; HDL-C, high-density lipoprotein cholesterol; LDL-C, low-density lipoprotein cholesterol

As shown in Table 1, In females, participants with longer sedentary time were significantly older than those in the < 4 h/day group, whereas in males no significant age difference was observed across sedentary categories. In both sexes, longer sedentary time was associated with higher likelihood of being a professional, higher educational attainment, a family history of metabolic diseases, lower levels of PA and shorter sleep durations (all *p*_for trend_ < 0.05). Higher prevalences of hypertension, dyslipidemia, and NAFLD were observed in participants with longer sedentary time compared to those with < 4 h/day sedentary time in both sexes.

In males, compared to those with < 4 h/day of sedentary time, participants with longer sedentary time exhibited higher levels of TC, TG and HDL-C, along with lower SBP. However, no significant differences were observed in BMI, FG, DBP, or LDL-C. However, a different profile was observed in females, where higher FG, TC, TG and HDL-C but lower BMI, SBP and DBP were observed in individuals with longer sedentary time compared to those with < 4 h/day of sedentary time. Nevertheless, no significant differences were detected in LDL-C among the four groups.

### Sex difference in the prevalence of metabolic Multimorbidity according to sedentary time category

The prevalence of metabolic multimorbidity was 26.47% (95% CI:23.92–29.02%), 25.64% (95% CI:24.11–27.17%), 25.22% (95% CI:24.10–26.34.10.34%), and 25.44% (95% CI:24.64–26.24%) for < 4, 4–<6, 6–<8, and ≥ 8 h/day sedentary time among the total population (Fig. 2b). Although females exhibited a higher proportion of (≥ 4 h/day) sitting time compared to males, the prevalence of metabolic multimorbidity was significantly higher in males (40.72%, 95% CI: 39.78–41.66%) than in females (10.35%, 95% CI: 9.78–10.92%) (*P* < 0.001). The prevalence of metabolic multimorbidity among males with daily sitting times of < 4, 4–<6, 6–<8, and ≥ 8 h/day was 38.00% (95% CI: 34.24–41.76%), 38.80% (95% CI: 36.49–41.11%), 40.21% (95% CI: 38.43–42.00.43.00%), and 41.93% (95% CI: 40.61–43.25%), respectively; whereas among females, the corresponding rates for the same sitting time categories were 12.06% (95% CI: 9.24–14.88%), 10.4% (95% CI: 8.79–11.91%), 10.15% (95% CI: 9.05–11.26%), and 10.32% (95% CI: 9.55–11.09%) (Fig. 2c).

### Association between sedentary time and metabolic multimorbidity

As shown in Supplementary Table 1, among the total population, both sedentary time and sex were associated with metabolic multimorbidity after fully adjusting for covariates (Model 4). The ORs (95% CIs) were 1.07 (0.91–1.27), 1.17 (1.00–1.38.00.38), and 1.21 (1.03–1.41) for 4-<6, 6-<8, and ≥ 8 h/day sedentary time, respectively. The OR (95% CI) for sex was 5.45 (5.00–5.93.00.93).​ The interaction between sex and sedentary time was statistically significant​ (*p* for interaction = 0.029), indicating sex differences in the associations between sedentary time and metabolic multimorbidity.

Table 2 shows the adjusted sex-specific associations between sedentary time and metabolic multimorbidity. In males, longer sedentary time was significantly associated with increased odds of metabolic multimorbidity. In the unadjusted regression model (model 1), compared to the reference group (< 4 h/day), the ORs (95% CIs) were 1.03 (0.86–1.25), 1.10 (0.92–1.31), and 1.18 (1.00–1.39.00.39) for 4-<6, 6-<8, and ≥ 8 h/day sedentary time, respectively. After adjusting for age, occupation, marital status, and education level (model 2), although 4-<6 h/day of sedentary time did not reach statistical significance, 6-<8 and ≥ 8 h/day sedentary time remained significantly associated with an increased risk of metabolic multimorbidity. The ORs declined slightly but remained significant in model 3, where family history of metabolic diseases was additionally adjusted. After further adjustment for smoking status, alcohol consumption, PA level, and sleep duration, the ORs (95% CIs) for 4-<6, 6-<8, and ≥ 8 h/day sedentary time were attenuated to 1.09 (0.90–1.32), 1.22 (1.01–1.47), and 1.26 (1.05–1.50), respectively, suggesting that longer sedentary time was positively associated with metabolic multimorbidity with a categorical dose-response trend (*P* for trend = 0.019).

**Table 2:** The ORs (95%CI) for the sex-specific association between sedentary time and metabolic Multimorbidity in young adults

	Sedentary time				*P*_for trend_
	< 4 h/day	4-<6 h/day	6-<8 h/day	≥ 8 h/day	
No. of males	642	1714	2885	5354	
No. of cases in males	244	665	1160	2245	
Model 1^a^	Ref	1.03 (0.86, 1.25)	1.10 (0.92, 1.31)	1.18 (1.00, 1.39)	0.043
Model 2^b^	Ref	1.11 (0.92, 1.35)	1.29 (1.07, 1.55)	1.41 (1.18, 1.68)	< 0.001
Model 3^c^	Ref	1.08 (0.89, 1.31)	1.24 (1.03, 1.49)	1.31 (1.10, 1.57)	0.001
Model 4^d^	Ref	1.09 (0.90, 1.32)	1.22 (1.01, 1.47)	1.26 (1.05, 1.50)	0.019
No. of females	514	1468	2867	6000	
No. of cases in females	62	151	291	619	
Model 1^a^	Ref	0.84 (0.61, 1.15)	0.82 (0.62, 1.10)	0.84 (0.64, 1.11)	0.624
Model 2^b^	Ref	1.11 (0.80, 1.54)	1.19 (0.88, 1.63)	1.29 (0.96, 1.75)	0.209
Model 3^c^	Ref	1.08 (0.78, 1.50)	1.13 (0.83, 1.54)	1.20 (0.89, 1.62)	0.516
Model 4^d^	Ref	1.08 (0.78, 1.50)	1.12 (0.82, 1.53)	1.17 (0.86, 1.58)	0.711

^a^ Model 1 was univariate model. ^b^ Model 2 was adjusted for age, occupation, marital status and education level. ^c^ Model 3 was further adjusted for family history of metabolic diseases. ^d^ Model 4 was further adjusted for smoking status, alcohol intake, PA level and sleep time

In contrast to males, the association between sedentary time and metabolic multimorbidity was not statistically significant in females across all regression models. In the unadjusted regression model (model 1), the crude ORs (95% CIs) for prolonged sedentary time were 0.84 (0.61–1.15), 0.82 (0.62–1.10), and 0.84 (0.64–1.11) for 4-<6, 6-<8, and ≥ 8 h/day of sedentary time, respectively, compared with < 4 h/day sedentary time. However, the ORs (95% CIs) increased to 1.11 (0.80–1.54), 1.19 (0.88–1.63), and 1.29 (0.96–1.75) after adjustment for age, occupation, marital status, and education level in model 2. The ORs attenuated slightly but remained non-significant after further adjustment for family history of metabolic diseases in model 3. In the fully adjusted model (model 4), the ORs (95% CIs) for 4-<6, 6-<8, and ≥ 8 h/day sedentary time were 1.08 (0.78–1.50), 1.12 (0.82–1.53), and 1.17 (0.86–1.58), respectively. Although the association between sedentary time and metabolic multimorbidity was not statistically significant in females, the ORs were all greater than the reference level, particularly for 6-<8 and ≥ 8 h/day sedentary time.

### Subgroup analysis

**Fig. 3:**
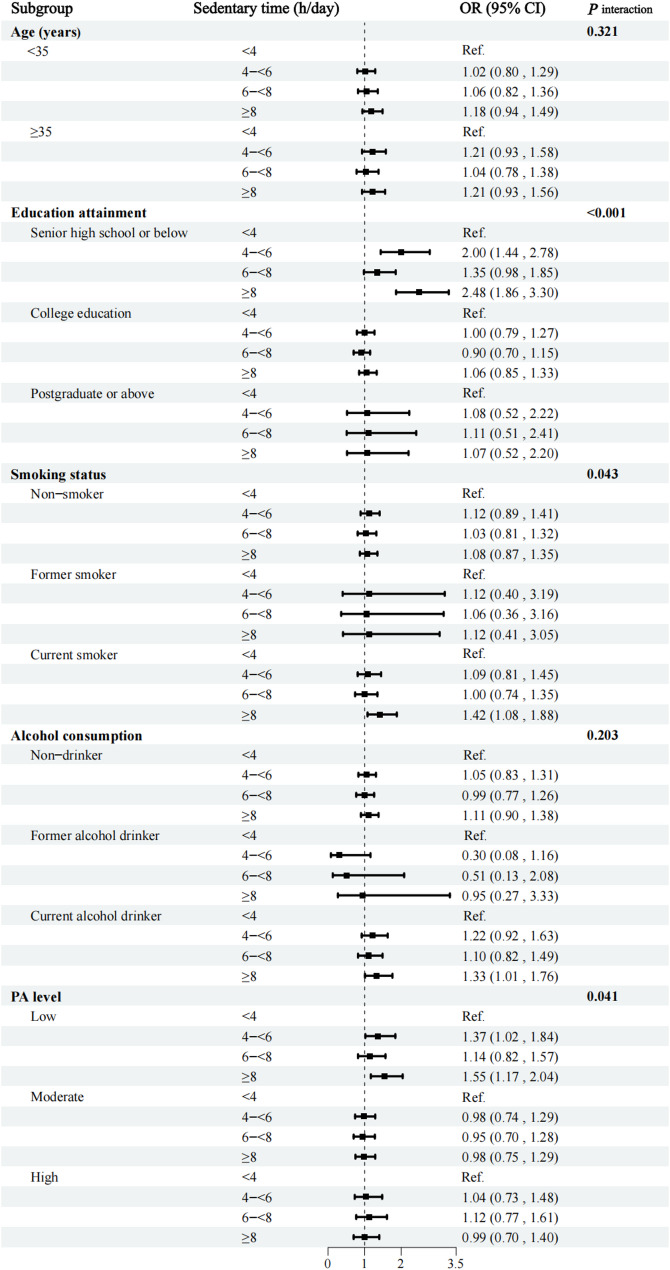
Association between sedentary time and metabolic multimorbidity across subgroups. PA, physical activity level; OR, odds ratios; 95% CI, 95% confidence intervals. The model of stratified analysis was adjusted for age, occupation, marital status and education level, family history of metabolic diseases, smoking status, alcohol intake, PA level and sleep time at baseline, excluding the variable for stratification

To examine whether age, smoking status, alcohol intake, or PA level interacted with sedentary time in the detected associations, we included these variables in the fully adjusted logistic regression model and performed subgroup analyses by the variables with statistically significant interaction terms. In this study, the association between sedentary time and metabolic multimorbidity was more pronounced in males than in females. Therefore, we performed the stratified analysis in males, and the results are shown in Fig. 3. We observed significant interactions between sedentary time and education level (*P* for interaction < 0.001), smoking status (*P* for interaction = 0.043), and PA level (*P* for interaction = 0.041). The association between sedentary time and metabolic multimorbidity was more pronounced among men with lower educational attainment, smoking habits, or lower PA levels.

### Sensitivity analyses

To assess the robustness of our results, we excluded 803 men and 81 women with clinical diagnoses of hypertension, diabetes, hyperlipidemia, or NAFLD, or those receiving pharmacological treatment for these metabolic disorders at baseline, to reduce potential post-diagnostic lifestyle modifications. We then conducted fully adjusted logistic regression analyses to reassess the associations of sedentary time with metabolic multimorbidity. The findings remained robust, with the ORs for 6-<8 h/day and ≥ 8 h/day of sedentary time slightly increasing to 1.23 (95% CI 1.02–1.49) and 1.29 (95% CI 1.08–1.56), respectively, in men. The association between sedentary time and metabolic multimorbidity remained statistically non-significant in women.

## Discussion

To the best of our knowledge, this is the first study to investigate sex differences in the association between sedentary time and early-onset metabolic multimorbidity among a large sample of young adults. Our analysis revealed distinct sex-specific patterns. Specifically, longer sedentary time (≥ 4 h/day) was positively associated with increased odds of early-onset metabolic multimorbidity in men, whereas no significant association was observed in women. Furthermore, a categorical dose-response correlation was identified in males. Compared with a reference of < 4 h/day, men with 6-<8, and ≥ 8 h/day of sedentary time had 22% and 26% higher odds of metabolic multimorbidity, respectively. Additionally, the association between sedentary time and early-onset metabolic multimorbidity in men was modified by educational attainment, smoking status, and PA level. The strong, dose-response association between longer sedentary time and metabolic multimorbidity observed specifically in young men highlights this subgroup as a priority for public health action. Our results suggest that interventions for this population should move beyond general advice.

Although the association between sedentary behavior and metabolic risk is well established, research examining biological sex as a potential effect modifier in this association, especially among young adults, remains sparse. Our findings provide compelling epidemiological evidence that young men may be more susceptible to the deleterious metabolic effects of prolonged sedentary behavior compared with their female counterparts of similar age. We observed a similar proportion of participants reporting ≥ 4 h of daily sedentary time between males (93.94%) and females (95.26%). However, the prevalence of metabolic multimorbidity exhibited marked disparities between the sexes. Contrary to findings in general adult and elderly populations, men in our cohort of young adults demonstrated a stronger association with these detrimental impacts. A meta-analysis incorporating 4 prospective cohort studies and 22 cross-sectional studies, involving 105,239 participants and 16,185 cases of metabolic syndrome (MetS), revealed that both total sedentary time and screen time were associated with a higher risk of MetS. Notably, the observed association patterns varied by sex rather than age, with women showing a disproportionately higher risk elevation compared to men [35]. Similarly, among highly active older adults in Canada, the association between longer sedentary time and metabolic risk was independent of sex [36]. In contrast, our study identified marked sex-specific differences in these associations during young adulthood, patterns which diverged significantly from those observed in older populations. Such sex-differentiated findings carry crucial implications for developing targeted guideline recommendations on sedentary behavior management and metabolic syndrome prevention.

The observed sex difference may be partially attributable to the potential protective effects of estrogen in premenopausal women. Estrogen is known to promote energy homeostasis and improve insulin sensitivity, which could theoretically counteract the metabolic risks associated with longer sedentary time. Compared with age-matched males, premenopausal females with a normal menstrual history have higher levels of 17β-estradiol (E2), the main circulating estrogen. E2 promotes energy homeostasis, improves body fat distribution, ameliorates insulin resistance, enhances insulin sensitivity, improves β-cell function, and reduces inflammation [37]. Therefore, the protective effects of estrogen in young women may counteract the metabolic risks associated with longer sedentary time, whereas sedentary behavior appears to have greater clinical predictive value for assessing metabolic multimorbidity risk in young men.

Previous studies on metabolic diseases have primarily focused on the elderly population, as it is commonly believed that metabolic disorders tend to coexist in middle-aged and older adults [38, 39]. Consequently, data on metabolic multimorbidity in young populations remain scarce. Our study revealed a relatively high prevalence of metabolic multimorbidity (25.35%) among individuals aged ≤ 45 years, with particularly elevated rates in men (40.72%) compared to women (10.35%). To our knowledge, no prior studies have specifically investigated metabolic multimorbidity as a primary outcome in this demographic. Notably, the prevalence observed in young men in our study is comparable to that of general multimorbidity (which often includes metabolic diseases among other chronic conditions) in older populations reported elsewhere [40, 41]. This early-onset trend is consistent with findings from previous age-stratified epidemiological studies [42, 43] and has been linked to elevated health risks, including increased susceptibility to cancer [12] and premature mortality [13]. Therefore, our findings underscore that metabolic multimorbidity in young adults—characterized by an unexpectedly high prevalence at a relatively early life stage—represents a critical public health issue warranting further investigation.

In our study, the vast majority of participants (94.61%) reported ≥ 4 h of daily sedentary time, a finding consistent with previous studies demonstrating the high prevalence of sedentary behavior among young adults [44]. Research suggests that fast-paced work and study schedules, coupled with increasingly urbanized lifestyles, contribute to high sedentary time in this demographic [15]. Nevertheless, the prolonged sedentary time observed in our sample may be partly attributable to the high proportion of participants with a college or higher education and predominantly sedentary occupations. Another potential explanation involves the low physical PA level observed, as over one-third of participants (33.66%) did not meet the 2020 World Health Organization (WHO) recommendations [45]. Substantial evidence indicates an inverse association between sedentary time and PA, suggesting that sedentary behavior may displace time otherwise available for PA [46].

​.

Our findings suggest a particularly strong association between sedentary time and metabolic multimorbidity in young adults. In this group, prolonged sedentary behavior may represent a primary risk factor for metabolic multimorbidity, likely because the influence of other contributing factors remains less pronounced during this life stage. As reviewed in the literature, metabolic multimorbidity arises from multifactorial etiologies, encompassing socioeconomic, lifestyle, and metabolic determinants [47–51]. Age-related variations in socioeconomic status, lifestyle behaviors, and metabolic function may contribute to differential susceptibility across age groups [52]. Notably, compared to genetic and infectious factors, sedentary behavior—an early-life exposure prevalent since adolescence—may play a pivotal role in the early development of metabolic multimorbidity among younger individuals [53]. Notably, the association between sedentary time and metabolic multimorbidity was modified by education level, smoking status, and PA. These factors are known to correlate with both chronic sedentary behavior and metabolic risk. Individuals with lower education and current smoking tend to have higher levels of prolonged sitting and poorer health behaviors, while higher PA may mitigate the adverse effects of sedentary time. Thus, the metabolic impact of sedentary behavior may be partly explained by these coexisting lifestyle and socioeconomic factors. Our findings suggest that comprehensive lifestyle interventions during early life stages may yield substantial health benefits.

Notably, the association between sedentary time and metabolic multimorbidity was modified by education level, smoking status, and PA. These factors are known to correlate with both chronic sedentary behavior and metabolic risk. Individuals with lower education and current smoking tend to have higher levels of longer sitting time and poorer health behaviors, while higher PA may mitigate the adverse effects of sedentary time. Thus, the metabolic impact of sedentary behavior may be partly explained by these coexisting lifestyle and socioeconomic factors.

​As an epidemiological study, our data describe associations of sedentary behavior with metabolic multimorbidity and cannot elucidate underlying biological mechanisms. The observed strong association, particularly in men, may be explained by its impact on fundamental metabolic pathways. Sedentary behavior is known to induce peripheral insulin resistance and suppress lipoprotein lipase activity, disrupting both glucose and lipid metabolism. This provides a plausible pathway for the development of the core components of metabolic multimorbidity we observed, including T2D, dyslipidemia (e.g., elevated TG), hypertension and obesity [18, 54–56]. Intervention trials have demonstrated that breaking up prolonged sitting with standing or walking lowers postprandial glucose, insulin, and lipid concentrations [57]. Nevertheless, the adverse metabolic consequences of prolonged sedentary time are not fully counteracted by high levels of PA [36]. The sexual dimorphism in these associations could stem from inherent differences in body fat distribution and sex hormone regulation of energy metabolism, making men potentially more susceptible to the deleterious metabolic effects of chronic muscle disuse.

Key strengths of this study include its large sample of young adults. This robust sample size enabled stratified analyses to examine the specific associations between sedentary time and metabolic multimorbidity. Furthermore, the data were sourced from the BTH-MEC, a natural population cohort. The age and sex distribution of our study population is comparable to that of the general Chinese adult population [58], supporting the generalizability of our findings. An additional strength is the comprehensive data collection on demographics, medical history, and lifestyle factors. These variables were adjusted for in the analysis as potential confounders, thereby strengthening the reliability of our results.

However, several limitations of this study should be acknowledged. First, due to the cross-sectional design, causality between sedentary time and metabolic multimorbidity cannot be inferred. Reverse causation is also possible, individuals with metabolic disorders may have adopted a more sedentary lifestyle due to fatigue. Second, the IPAQ-SF was chosen for its feasibility in a large epidemiological sample and to allow comparison with previous studies. Sedentary time was self-reported, making it susceptible to recall bias and social desirability bias, and reporting accuracy may vary by sex, education, and occupation. Differential misclassification of sedentary time remains a potential source of bias in the present study [59]. Future studies should employ objective measures (e.g., accelerometry) to enhance measurement precision. Third, in this study, we focused primarily on the association between current sedentary time and prevalent metabolic multimorbidity. However, duration of exposure to a sedentary lifestyle was not directly measured in our dataset. It is plausible that the duration of sedentary lifestyle could modify metabolic risk, and our cross-sectional design cannot disentangle this temporal relationship. In future prospective studies, collecting detailed histories of sedentary behavior (e.g., years in occupations with prolonged sitting, leisure-time screen exposure since adolescence) would allow direct examination of this hypothesis. Fourth, the use of a composite definition of metabolic multimorbidity, defined as the co‑occurrence of any two of five conditions. These conditions differ in severity, pathophysiological mechanisms, and potential for reversibility, and treating all combinations as equivalent may obscure meaningful heterogeneity. This approach may also overestimate prevalence, particularly in young adults in whom subclinical or borderline cases are common. Future studies should explore disease‑specific combinations and severity levels to refine the clinical interpretation of metabolic multimorbidity in this age group. Fifth, although we adjusted for a range of potential confounders, residual or unmeasured confounding (e.g., from dietary habits, pregnancy or perimenopause status, psychological symptoms) cannot be ruled out. Finally, as participants were recruited from the China BTH-MEC cohort, caution should be exercised when generalizing the observed sex-specific associations to other populations.

## Conclusions

Our study reveals distinct sex differences in the association between sedentary time and early-onset metabolic multimorbidity, with sedentary time showing categorical dose-response association with the odds of metabolic multimorbidity in young men, but demonstrating no significant association in young women. These findings highlight the potential for reducing metabolic multimorbidity risk through early interventions targeting sedentary behavior in young men. Such targeted strategies are likely to be more feasible and effective than broad, non-specific recommendations, and implementing them early in adulthood could help curb the progression toward clinical metabolic diseases. Furthermore, the findings underscore the critical importance of considering sex-specific differences in both the design of intervention strategies and the expected health benefits.

## Supplementary Information

Supplementary Material 1.

## Data Availability

Data supporting the findings of this study are available in the main text and supplemental materials. Individual-level data can be obtained from the corresponding author upon reasonable request.
