# Supplementary material for: Sex differences in the impact of sedentary time on early-onset metabolic multimorbidity: evidence from a study of over 20,000 young adults
Source: Biol Sex Differ. 2026 Jan 28;17:36. doi: 10.1186/s13293-026-00831-x (PMC12922324; doi:10.1186/s13293-026-00831-x)
Supplement: Supplementary file 1 — Supplementary Material 1. [file 13293_2026_831_MOESM1_ESM.docx]

**Supplementary table 1.** Odds ratios (95% CI) for associations between study variables and metabolic multimorbidity in young adults

| Variables | Total sample | | Men | | Women | |  |
| --- | --- | --- | --- | --- | --- | --- | --- |
|  | OR (95%CI) | *P* | OR (95%CI) | P | OR (95%CI) | *P* |  |
| Sedentary duration | | | | | | |  |
| < 4 hours/day | 1 |  | 1 |  | 1 |  |  |
| 4–6hours/day | 1.07(0.91,1.27) | 0.423 | 1.09(0.90,1.32) | 0.396 | 1.08(0.78,1.50) | 0.653 |  |
| 6–8 hours/day | 1.17(1.00,1.38) | 0.052 | 1.22(1.01,1.47) | 0.039 | 1.12(0.82,1.53) | 0.488 |  |
| ≥ 8 hours/day | 1.21(1.03,1.41) | 0.018 | 1.26(1.05,1.50) | 0.013 | 1.17(0.86,1.58) | 0.324 |  |
| Sex | | | | | | |  |
| Female | 1 |  | - | - | - | - |  |
| Male | 5.45(5.00,5.93) | <0.001 | - | - | - | - |  |
| Age group (years) | | | | | | |  |
| 18–25 | 1 |  | 1 |  | 1 |  |  |
| 26–30 | 1.31(1.09,1.56) | 0.004 | 1.21(0.98,1.49) | 0.078 | 1.60(1.10,2.34) | 0.015 |  |
| 31–35 | 1.69(1.40,2.04) | <0.001 | 1.53(1.23,1.90) | <0.001 | 2.24(1.53,3.29) | <0.001 |  |
| 36–40 | 2.18(1.80,2.64) | <0.001 | 2.00(1.60,2.49) | <0.001 | 2.79(1.90,4.10) | <0.001 |  |
| 41-45 | 2.47(2.03,3.00) | <0.001 | 2.11(1.68,2.65) | <0.001 | 3.55(2.40,5.25) | <0.001 |  |
| Occupation | | | | | | |  |
| Professional | 1 |  | 1 |  | 1 |  |  |
| Civil servants | 0.92(0.84,1.00) | 0.048 | 0.95(0.85,1.05) | 0.298 | 0.84(0.71,0.99) | 0.041 |  |
| Other | 1.15(1.05,1.25) | 0.002 | 1.20(1.08,1.33) | 0.001 | 1.06(0.91,1.24) | 0.447 |  |
| Marital status | | | | | | |  |
| In a current marriage | 1 |  | 1 |  | 1 |  |  |
| Single | 0.73(0.65,0.81) | <0.001 | 0.71(0.63,0.81) | <0.001 | 0.78(0.63,0.97) | 0.025 |  |
| Divorced/widowed | 1.30(0.98,1.73) | 0.068 | 1.24(0.87,1.76) | 0.241 | 1.39(0.88,2.21) | 0.161 |  |
| Education | | | | | | | |
| High school or below | 1 |  | 1 |  | 1 |  |  |
| College/university | 0.79(0.71,0.88) | <0.001 | 0.87(0.77,0.98) | 0.022 | 0.58(0.46,0.73) | <0.001 |  |
| Postgraduate/above | 0.53(0.47,0.61) | <0.001 | 0.61(0.52,0.71) | <0.001 | 0.36(0.28,0.47) | <0.001 |  |
| Family history of metabolic diseases | | | | | | | |
| No | 1 |  | 1 |  | 1 |  |  |
| Unknown | 0.91(0.77,1.07) | 0.251 | 0.89(0.74,1.07) | 0.207 | 1.02(0.73,1.41) | 0.919 |  |
| Yes | 1.57(1.46,1.68) | <0.001 | 1.53(1.41,1.66) | <0.001 | 1.67(1.46,1.91) | <0.001 |  |
| Smoking | | | | | | |  |
| Never | 1 |  | 1 |  | 1 |  |  |
| Former smoker | 1.09(0.89,1.35) | 0.411 | 1.12(0.90,1.38) | 0.307 | 1.05(0.35,3.11) | 0.931 |  |
| Yes | 1.30(1.19,1.42) | <0.001 | 1.32(1.20,1.44) | <0.001 | 1.37(0.90,2.09) | 0.148 |  |
| Alcohol drinking | | | | | | |  |
| Never | 1 |  | 1 |  | 1 |  |  |
| Former alcohol user | 1.21(0.86,1.70) | 0.283 | 1.05(0.74,1.50) | 0.789 | 4.46(1.82,10.96) | 0.001 |  |
| Yes | 1.13(1.04,1.22) | 0.005 | 1.13(1.04,1.23) | 0.006 | 1.11(0.83,1.48) | 0.498 |  |
| Physical activity | | | | | | |  |
| Low | 1 |  | 1 |  | 1 |  |  |
| Moderate | 1.05(0.97,1.13) | 0.25 | 1.02(0.92,1.12) | 0.742 | 1.11(0.97,1.28) | 0.132 |  |
| High | 0.65(0.59,0.72) | <0.001 | 0.62(0.56,0.70) | <0.001 | 0.72(0.59,0.87) | 0.001 |  |
| Sleep duration | | | | | | |  |
| 4–6 hours/day | 1 |  | 1 |  | 1 |  |  |
| 7–8 hours/day | 0.91(0.82,1.02) | 0.092 | 0.94(0.83,1.06) | 0.3 | 0.84(0.68,1.04) | 0.104 |  |
| 9–11 hours/day | 0.88(0.76,1.01) | 0.063 | 0.90(0.76,1.06) | 0.21 | 0.82(0.64,1.07) | 0.141 |  |

Results from the fully adjusted logistic regression model, which included all relevant variables.
